# Not just there by chance: a qualitative study on continuity and quality of spiritual care in paediatric palliative care

**DOI:** 10.1007/s00431-026-07182-0

**Published:** 2026-06-23

**Authors:** L.oes Berkhout, Marije P. Hennus, Anne C. E. Helms, Marjorie A. C. P. de Man, Wim Smeets, Adri Spelt, Marijke C. Kars

**Affiliations:** 1 Department of Spiritual Care, University Medical Center Utrecht, 3508 AB Utrecht, The Netherlands; 2 Paediatric Intensive Care, Wilhelmina Children’s Hospital, University Medical Center Utrecht, Utrecht, The Netherlands; 3 Association of Spiritual Caregivers (VGVZ), Zeist, The Netherlands; 4 Paediatric Intensive Care, University Medical Center Utrecht/Wilhelmina Children’s Hospital, Utrecht, The Netherlands; 5 Department of Spiritual and Pastoral Care, Radboud University Nijmegen Medical Centre, Nijmegen, The Netherlands; 6 Department of Spiritual Care, University Medical Center Groningen, Groningen, The Netherlands; 7 Julius Center for Health Sciences and Primary Care, Center of Expertise in Palliative Care Utrecht, University Medical Center Utrecht, Utrecht, The Netherlands

**Keywords:** Spiritual care, Paediatric palliative care, Continuity, Interdisciplinary consultation

## Abstract

Children with life-limiting conditions and their families face profound existential and spiritual challenges throughout the paediatric palliative care (PPC) trajectory. While spiritual care (SC) is a recognised component of PPC, its continuity and quality across care settings remain underexplored. This study examined how SC can be organised and safeguarded across the PPC continuum, from the perspectives of parents, healthcare professionals, and spiritual care providers. An exploratory qualitative study was conducted using multidisciplinary peer consultation groups. Two groups met online in 2022–2023, discussing six PPC cases over seven sessions. Participants included spiritual care professionals, clinicians from hospital and home settings, and parents. Data sources included audio-recorded transcripts, structured case descriptions, and field notes. A modified QUAGOL approach was used for narrative-informed thematic analysis. Coding was supported with team discussions. Continuity of care (relational, informational, and managerial) informed interpretation as a sensitising framework. Two themes were identified: (1) challenges in ensuring continuity of spiritual care, including fragmented referrals, lack of coordination, and cultural disconnects; and (2) defining and delivering quality SC, emphasising relational consistency, cultural and linguistic fit, and interprofessional collaboration. Parents valued providers who “knew their story”, while professionals stressed clearer referral pathways and role alignment. Gaps in coordination and information transfer constrained support across transitions.

*Conclusion*: Continuity of spiritual care in PPC is not merely a logistical task but a relational and interpretive process. Embedding SC into proactive care planning and interprofessional practice may improve coordination, enhance cultural responsiveness, and support families throughout the illness trajectory.

**Table:** 

**What is Known:** • *Spiritual care is an essential dimension of paediatric palliative care but is often fragmented across settings. Traditionally, SC has been hospital-centred.* • *Families frequently lack consistent support during transitions between hospital, home, and community care.*
**What is New:** • *This study suggests that continuity in spiritual care relies on sustained relationships, cultural attunement, and clear coordination.* • *Embedding spiritual care in proactive planning may support consistency and family-centred support throughout the illness trajectory.*

**What is Known:**

• *Spiritual care is an essential dimension of paediatric palliative care but is often fragmented across settings. Traditionally, SC has been hospital-centred.*

• *Families frequently lack consistent support during transitions between hospital, home, and community care.*

**What is New:**

• *This study suggests that continuity in spiritual care relies on sustained relationships, cultural attunement, and clear coordination.*

• *Embedding spiritual care in proactive planning may support consistency and family-centred support throughout the illness trajectory.*

## Introduction

Children with life-limiting conditions and their families often face profound existential and spiritual challenges as they navigate paediatric palliative care (PPC)(2) []. Palliative care is defined by the WHO as an approach that improves the quality of life of patients and their families facing life-threatening illness by preventing and relieving suffering through early identification, assessment, and treatment of pain and other physical, psychosocial, and spiritual problems [21]. Spiritual care (SC) is generally understood as addressing existential and spiritual needs of patients and families, religious or otherwise [4]. Continuity and quality of SC across healthcare settings, however, remain persistently difficult to achieve. Families frequently move between home, regional hospitals, and academic centres, creating gaps in SC access and coordination [1, 18].

Traditionally, spiritual care in PPC has been hospital-centred, often initiated during acute admissions or clinical turning points such as diagnosis, a shift to palliative intent, or end-of-life care [2, 9, 14]. In recent years, spiritual care has also become increasingly available through community-based spiritual caregivers in primary care settings; however, access, visibility, and continuity across settings remain variable. As more children live longer with complex conditions and receive care predominantly at home, new challenges emerge in ensuring existential and spiritual support that is context-sensitive and consistent across healthcare settings [7, 17, 20]. Transitions between care settings further increase the risk of fragmentation.

Spiritual care itself has evolved from a narrow religious focus to a broader engagement with meaning-making, existential questions, and personal values [15]. Families living with a child’s gradual decline often grapple with anticipatory grief, uncertainty, and the search for meaning that extends well beyond clinical encounters [11, 12]. These needs may remain unrecognised, particularly in community settings where awareness of SC and established referral pathways are often lacking [19].

This study is guided by continuity of care and meaning-making theory. We draw on Haggerty et al.’s continuity framework [8], distinguishing relational continuity (being known over time), informational continuity (families’ narratives and values travelling across settings), and managerial continuity (coordinated care and clear responsibility and referral pathways). Meaning-making theory conceptualises spiritual care as supporting families’ interpretation of, and coping with, existential questions across the illness trajectory [15]. Together, these perspectives highlight that continuity in spiritual care is not only organisational but also relational and interpretive.

Accordingly, this study aimed to examine how continuity and quality of spiritual care can be organised across the paediatric palliative care continuum. While previous qualitative studies have described families’ spiritual needs and experiences, less is known about how spiritual care is coordinated and sustained across transitions between hospital and home care. Using real-world cases discussed in multidisciplinary peer consultation groups, this study adopts a continuity-of-care perspective to identify challenges in recognising existential needs, in initiating spiritual care, and in maintaining coordination across care settings.

## Materials and methods

### Study design

This exploratory qualitative study aimed to investigate how continuity and quality of spiritual care (SC) for children with life-limiting conditions and their families are experienced by families and professionals throughout the paediatric palliative care trajectory and across different care settings. We adopted a collaborative peer consultation approach to elicit practice-based insights from diverse stakeholders. This method, with roots in Dutch traditions of interprofessional reflection [16], was well suited to capturing situated knowledge.

### Setting and participants

Two multidisciplinary peer consultation groups met online in 2022–2023 (Table 3), facilitated by an experienced certified spiritual care supervisor trained in professional supervision and intervision methods within the Dutch spiritual care context. In the Netherlands, spiritual care providers are trained professionals who may have a general, humanistic, or religious educational background. Participants included SC professionals from primary and tertiary care, healthcare providers involved in PPC, and parents of children who had received palliative care. Recruitment took place via professional and national palliative care networks and spiritual care associations. Parents were eligible if they had recently cared for a child at home during the palliative phase.

### Data collection

Sessions followed an adapted “Task-Oriented Learning” model [16], focussing on SC transitions and experiences of continuity across settings (Table1). Each 2-h session comprised a structured case presentation, collaborative analysis, and group reflection. Cases were submitted by SC providers, clinicians, or parents using a standardised format (Table 1).

**Table 1:** Peer consultation: comparison of Van Kessel’s original model and the adapted model for this study

Original model (Van Kessel & Haan [16]	Adapted model for this protocol
Introductory round	Identical to original model
Review of previous session	Removed
Case presentation by participant	Case presentation by new external contributor
Explanation of focus/method	Identical to original model
Break	Identical to original model
Detailed description of the action process	Identical to original model
Self-reflection (on content)	Reflection on the continuity of the spiritual care process by the contributor
Exchange of reflections with other participants	Identical to original model
Answering the question: What do we learn from this?	Identical to original model
Session review	Identical to original model

Methodological quality drew on audio-recorded transcripts, structured case descriptions, background questionnaires, and field notes. These multiple layers of data enhanced the contextual richness of the analysis and helped capture both spoken and unspoken dynamics within the sessions. Across seven meetings, six cases were discussed; one case was revisited in both groups to compare perspectives across settings.

### Data analysis

Analysis was informed by QUAGOL [3], beginning with narrative case reconstructions that informed development of a coding tree. This tree was refined after a second narrative round, after which formal coding was conducted using NVivo.

Six researchers (LB, AH, MJM, AS, WS, MCK) independently familiarised themselves with the data; one researcher (LB) coded the full dataset, with iterative team discussions ensuring researcher triangulation and analytic rigour. NVivo (QSR International, v.12) was used for coding, comparison, and retrieval, which enabled systematic organisation of large volumes of text and facilitated thematic synthesis across sessions. Continuity of care (relational, informational, and managerial) was used only as a sensitising framework during interpretation. Coding and theme development proceeded inductively; themes were subsequently examined to explore how barriers and enablers related to recognising existential needs, initiating spiritual care, and maintaining coordination across care setting. Analytic memos were employed throughout the process to document decisions, emergent patterns, and points of interpretive tension [13].

### Ethical considerations

The study was approved by the Medical Ethics Committee of the University Medical Center Utrecht (research proposal number 22/485) and deemed exempt from the Dutch Medical Research Involving Human Subjects Act (WMO), given its minimal risk. This study was performed in accordance with the principles of the Declaration of Helsinki. Informed consent was obtained from all participants.

### Reflexivity

The research team acknowledged the potential influence of their professional backgrounds on interpretation. The lead researcher (LB), an experienced SC provider in PPC, facilitated recruitment and ensured contextual sensitivity but also posed a risk of insider bias. This was mitigated by engaging a multidisciplinary research team with both SC and clinical expertise. Team members discussed transcripts and codes collaboratively, to reflect on positionality and ensure transparency throughout the process.

## Results

### Participants and setting

Participants included spiritual care professionals (SCs), healthcare professionals (HCPs) involved in paediatric palliative care (PPC), and parents of children who had received such care. Participant characteristics and case types are detailed in Tables 2 and 3. Group 1 comprised two home care nurses, two SCs, and two bereaved parents. Group 2 included a paediatrician, a home care nurse, two SCs, and two parents, one of whom withdrew after two sessions due to their child’s recent death.

**Table 2:** Characteristics of the presented cases

Case	Group	Presenter/diagnosis	Fam. View	Age (year)	Residence/Transition	Deceased	SC
1.1	1	Parents/patient/genetic defect	Christian	8	Home ↔ university hospital	No	Yes, university hospital
1.2	1	Parents/prematurity	n/a	0	Hospital → home	Yes	Yes, home
1.3	1	Social worker/cancer	Islamic	7	Specialised hospital	Yes	No
2.1	2	Spiritual caregiver/cancer	Islamic	7	Specialised hospital-->Home	Yes	Yes, home
2.2	2	Nurse consultant/metabolic disease	n/a	16	Chronic care institution → university hospital	No	No
2.3	2	Spiritual caregiver/down syndrome and heart defect	n/a	6	Home → university hospital	Yes	Yes, university hospital
2.4	2	Mother/extreme prematurity and comorbidities	Roman Catholic	15	Home → university hospital	Yes	Yes, university hospital

*Fam. View* family’s religious/spiritual background, *Residence/Transition* main care setting and movements between settings, *SC* spiritual care involvement and primary setting, *n/a* not available or not applicable

**Table 3:** Participants’ characteristics

Group	Characteristic	Categories	*n*
Spiritual caregivers (***n*** = 4)	Affiliation	University hospital	2
		Private practice	1
		General hospital + private practice	1
	Religious background	Christian	3
		Buddhist	1
Healthcare professionals (***n*** = 4)	Profession	Home care nurse	3
		Physician (paediatrician)	1
	Years of experience	< 5 years	1
		> 15 years	3
Parents (***n*** = 4)	Relation to child	Mother	3
		Father	1
	Diagnosis of child	Malignancy	2
		Non-malignancy	2

Seven online peer consultation sessions were conducted: group 1 met three times and group 2 four times. Each session lasted approximately 2 h and the series per group was facilitated by the same supervisor, with the observing researcher (LB) present to ensure consistency. Sessions were held online, to support geographic accessibility. All participants attended at least one session, with attendance ranging from six to nine participants.

Across the seven meetings, six unique cases were discussed (Table 4). One case was intentionally revisited in both groups to compare perspectives across care settings.

**Table 4:** Characteristics of the case contributors

Session	Group 1	Group 2
1	Spiritual caregiver (primary care)	Parents of a child in PPC
2	Spiritual caregiver (university hospital)	Recently bereaved parents
3	Parent who had recently lost a child	Medical social worker (specialised paediatric hospital)
4	Nurse specialist (university hospital)	–

### Thematic findings: continuity and quality of spiritual care

We identified two overarching domains shaping the experiences of families and professionals: challenges in ensuring continuity of spiritual care and defining and delivering quality spiritual care across paediatric palliative care settings.

#### Challenges in ensuring continuity of spiritual care

This theme predominantly reflects disruptions in managerial and informational continuity.

##### Initiation of support

Across both groups, participants described uncertainty about when and how spiritual care was introduced. Parents frequently perceived initial contact with an SC professional as serendipitous or vague. As one parent explained (1.2 R7):

“*Our contact with the Spiritual caregiver was, in fact, initiated by coincidence*”

This anecdotal emergence of support led to inconsistency. SC involvement often began during moments of acute distress, diagnosis, worsening prognosis, or end-of-life conversations, within hospital settings. Such moments were less clear in the home environment, where transitions are more diffuse, and the need for spiritual care can be prolonged yet subtler. Several professionals highlighted the tension between wanting to provide early support and the practical constraints surrounding more systematised referral processes, including limited availability of specialised paediatric spiritual care providers, lack of standardised referral triggers, and limited integration of spiritual care into existing communication pathways. This reactive model of SC initiation was widely seen as inadequate for addressing the chronic existential needs of families caring for a seriously ill child at home.

##### Referral pathways and recognition of needs

Whereas initiation of spiritual care was often reactive, this subsection focuses on uncertainty in recognising spiritual needs and responsibility for referral. Parents described difficulties articulating spiritual needs, noting they rarely used explicit spiritual language. Healthcare providers acknowledged this, adding that recognising existential suffering required both time and relational depth, resources often limited in day-to-day practice. One parent said (2.4 R):

“*With a clear (red. existential) question too, almost a cry for help: Why weren’t we able to have that guidance earlier.*”

Several SC professionals observed that implicit biases about a family’s perceived coping skills, cultural norms, or communication style sometimes hindered referral decisions. Moreover, SC dedicated to paediatrics was often embedded in tertiary care teams, making it less visible and accessible in primary care settings. Participants emphasised the need for shared understanding across providers about the scope of SC, the triggers for referral, and mechanisms for proactive outreach from tertiary care teams.

##### The role of the lead professional

Participants underscored the importance of a clearly designated “lead” person to coordinate SC across settings. Parents described feeling unsupported during transitions, particularly when moving from hospital to home care. As one HCP (2.1 M1) explained:

“*you actually need somebody working in different fields, connecting intramural with extramural and all the disciplines represented in both.*”

Professionals noted that assuming such a coordinating role requires knowledge of SC, the confidence to address existential concerns, and the ability to bridge communication gaps between institutions. Participants advocated for a care coordinator who not only ensures logistical handovers but also holds space for relational and existential continuity.

#### Defining and delivering quality spiritual care

This theme primarily reflects relational continuity, while also highlighting the importance of supportive managerial processes for sustaining quality SC.

##### Understanding the scope of spiritual care

Professionals in both groups agreed that spiritual care is broader than religious practice. SC encompasses meaning-making, value clarification, anticipatory grief, and navigating complex decisions. While all HCPs may touch upon these issues, participants emphasised the added value of trained SC professionals in addressing deeply personal and existential topics. One paediatrician (1.1 R4) noted:

“*of course I know them, so we have those kinds of conversations sometimes too*”

Still, HCPs recognised the limits of their expertise, particularly when spiritual distress was persistent or linked to unresolved trauma. SC professionals expressed willingness to collaborate and highlighted the need for role clarity and mutual respect to avoid fragmentation or duplication. Participants emphasised the importance of a shared understanding of the contribution of spiritual care within paediatric palliative care, as well as awareness of each other’s expertise and roles. Greater familiarity with spiritual care, clearer referral pathways, and mutual understanding across disciplines were considered important to support collaboration and facilitate timely involvement of specialised spiritual care when existential concerns arise.

##### Building and sustaining relationships

Continuity in SC, according to numerous participants, was tied to long-term relationships. Parents appreciated when SC professionals followed them across care transitions or maintained contact over time. However, participants of one case also noted that even if a single SC was unavailable throughout the entire trajectory, continuity could be achieved through shared documentation, team alignment, and consistent values-based communication. However, systemic barriers such as fragmented funding and institutional silos often impeded this vision. One participant (2.3 I) noted:

“*the movement up and down between those lines.... how is something like that conveyed…, how do you do that sort of thing and how do you decide when who gets in?*”

##### Cultural and linguistic fit

Families valued SC providers who “spoke their language”, both literally and culturally. Participants emphasised the need for culturally sensitive, trauma-informed spiritual support that acknowledges and reflects families’ values, beliefs, and experiences. A mother from a religious background (2.4, R) shared:

“*You could talk about matters of faith with the SC at the children’s hospital, but you could also share your concerns as a mother.*”

Nevertheless, several participants noted the limited diversity among SC professionals in non-urban areas. This mismatch created barriers for families who might otherwise benefit from culturally concordant care.

##### Professional collaboration and role boundaries

Whereas earlier sections highlighted continuity in relationships with families, participants also addressed the importance of interprofessional collaboration and role boundaries. Some home care nurses felt equipped to explore existential questions, whereas others expressed hesitation.

There was consensus that improving SC continuity required system-wide investment in role awareness, communication pathways, and relational trust among professionals. Participants advocated for shared care plans that include spiritual needs, accessible SC referral systems, and interprofessional training in existential dialogue. One participant (2.3 R) noted:

“*It means that we must remain constantly aware that our involvement, our responsibility, and our collaboration with one another — and with parents — are taking up an increasingly significant space. This requires a helicopter view in order to see other possibilities and perspectives.*”

## Discussion

This study explored how continuity and quality of spiritual care across the paediatric palliative care continuum are experienced by families and professionals. Using multidisciplinary peer consultation groups, we identified challenges in recognising existential needs, initiating spiritual care, and maintaining coordination across care settings. Parents often described spiritual support as fragmented or dependent on chance, while professionals reported uncertainty about roles, referral pathways, and cultural sensitivity. Interpreted through Haggerty’s framework, these challenges primarily reflected gaps in managerial continuity and informational continuity, which may undermine relational continuity.

Our findings align with previous research demonstrating that families’ existential needs evolve throughout the illness trajectory [9, 10]. In the Dutch PPC context, referrals to specialised paediatric spiritual care are still frequently initiated during hospital admissions or major clinical transitions (Meert et al.[14]; recognition and coordination of spiritual support in community settings may be less structured and more variable [2]. Although community-based spiritual care is increasingly available, participants described challenges in maintaining visibility, accessibility, and continuity across care transitions. These findings underscore the need for structured approaches that preserve continuity when care shifts across settings, particularly during transitions where coordination is limited.

Haggerty et al. [8] conceptualise continuity as including informational, managerial, and relational domains. Our findings highlight the interdependence of these domains: when coordination and transfer of families’ narratives falter, relational continuity becomes difficult to sustain. Many families described fractured support during transitions, and the absence of designated care coordinators further exacerbated feelings of abandonment and hindered trust-building. The study also adds to the literature on meaning-making and existential support in PPC [5, 15], which positions spiritual care as encompassing more than religious rites. Participants emphasised the importance of cultural identity, values, and everyday meaning-making. While HCPs recognised the importance of these conversations, they often felt ill-equipped to engage in them. This discomfort, echoed in earlier work by VGVZ [19] and [2], highlights the importance of fostering a shared understanding of spiritual care among healthcare professionals, including recognising existential concerns, understanding the potential contribution of spiritual care, and developing the language needed to discuss spiritual needs. Such shared mental models may support earlier recognition of spiritual distress and facilitate timely referral to specialised spiritual care when appropriate.

Our findings also highlight how continuity of care and meaning-making are closely interconnected in paediatric palliative care. Quality of spiritual care involves relational continuity, which allows families to feel known over time and supports shared narratives about illness, loss, and hope. Informational continuity helps preserve these narratives across settings, reducing the need for families to repeatedly reconstruct or defend their values and experiences. Conversely, disruptions in managerial continuity, such as unclear referral pathways or fragmented coordination, may interrupt meaning-making by creating narrative rupture, uncertainty, or a sense of abandonment during transitions. Continuity of spiritual care thus functions both as an organisational process and as a condition enabling families to sustain interpretive coherence.

Our findings align with Fahner et al. [6], who emphasise the importance of advance care planning in addressing physical and psychosocial dimensions as well as the spiritual dimension. Embedding spiritual care in such planning processes could improve continuity and ensure that families feel seen and supported during key transitions. Participants advocated for early, visible, and culturally appropriate involvement of SC providers. Cultural and linguistic fit emerged as particularly important for quality of SC, echoing earlier findings by Wiener et al. [20]. Quality in SC requires not only familiarity with families’ spiritual vocabulary but also understanding of their medical journey. While academic centres often offered greater diversity of SC providers, families in primary care settings sometimes experienced mismatches between their needs and available expertise. Organisational commitment to inclusion and cultural humility therefore remains essential.

Several implications emerge for clinical practice. All HCPs, including paediatricians, nurses, and members of multidisciplinary PPC teams, should be trained to recognise existential distress, initiate conversations about spirituality, and make timely referrals. Consistency of SC support across settings may be enhanced through shared care plans and joint handovers, particularly during transitions between hospital and home care. Culturally responsive care requires workforce diversification and education in structural competence. Interdisciplinary collaboration must be strengthened through role clarity, joint case reviews, and shared learning, supporting clinicians who are not spiritual care specialists. Finally, systemic barriers, including unclear reimbursement structures, limited SC visibility, and siloed information systems, must be addressed to enable seamless integration of SC into PPC. These strategies support managerial continuity, informational continuity, and relational continuity, thereby strengthening quality SC across settings and may also be relevant for paediatric populations with chronic complex care needs who experience prolonged trajectories and repeated care transitions.

Methodologically, this study benefitted from an adapted form of the QUAGOL approach [3], enabling iterative, narrative-informed analysis. Integrating perspectives of parents, SCs, and HCPs enriched understanding of real-world relational dynamics, while longitudinal peer consultation fostered reflection and co-learning. Limitations include the online format and limited representation of religious minorities and non-Western perspectives.

## Conclusion

This study underscores that continuity of spiritual care is more than a matter of structural coordination. It depends on sustained relationships, cultural and linguistic attunement, and mutual understanding across disciplines and settings. From this perspective, continuity of spiritual care supports not only coordinated service delivery, but also families’ ongoing meaning-making in the face of uncertainty, loss, and transition. Improving continuity requires attention to managerial, informational, and relational continuity. Embedding spiritual care into proactive planning and interdisciplinary routines, supported by training and aligned organisational structures, may enhance its accessibility, responsiveness, and value across the paediatric palliative care continuum. Ultimately, meeting families’ existential needs is not an optional enhancement, but an integral part of delivering humane, dignified, and person-centred care.

## Data Availability

Data were generated and analysed during the current study. To protect participant privacy and confidentiality, the qualitative data are reported in anonymised form within the manuscript but are not publicly available. Due to the sensitive nature of the discussions and the risk of indirect identification of participants, the full dataset cannot be shared.

## Abbreviations

HCP: Healthcare professional
PPC: Paediatric palliative care
SC: Spiritual care
VGVZ: Association of Spiritual Caregivers (in Dutch: Vereniging voor Geestelijk Verzorgers)
WMO: Medical Research Involving Human Subjects Act (in Dutch: Wet Medisch-wetenschappelijk Onderzoek)
